# Designing Driver Assistance Systems with Crossmodal Signals: Multisensory Integration Rules for Saccadic Reaction Times Apply

**DOI:** 10.1371/journal.pone.0092666

**Published:** 2014-05-06

**Authors:** Rike Steenken, Lars Weber, Hans Colonius, Adele Diederich

**Affiliations:** 1 Department of Psychology, European Medical School, Carl von Ossietzky Universität, Oldenburg, Germany; 2 OFFIS, Department for Transportation, Human-Centred Design, Oldenburg, Germany; 3 Department of Psychology, Cluster of Excellence “Hearing4all”, and Research Center Neurosensory Science, European Medical School, Carl von Ossietzky Universität, Oldenburg, Germany; 4 School of Humanities and Social Sciences, Jacobs University, Bremen, Germany; University of Muenster, Germany

## Abstract

Modern driver assistance systems make increasing use of auditory and tactile signals in order to reduce the driver's visual information load. This entails potential crossmodal interaction effects that need to be taken into account in designing an optimal system. Here we show that saccadic reaction times to visual targets (cockpit or outside mirror), presented in a driving simulator environment and accompanied by auditory or tactile accessories, follow some well-known spatiotemporal rules of multisensory integration, usually found under confined laboratory conditions. Auditory nontargets speed up reaction time by about 80 ms. The effect tends to be maximal when the nontarget is presented 50 ms before the target and when target and nontarget are spatially coincident. The effect of a tactile nontarget (vibrating steering wheel) was less pronounced and not spatially specific. It is shown that the average reaction times are well-described by the stochastic “time window of integration” model for multisensory integration developed by the authors. This two-stage model postulates that crossmodal interaction occurs only if the peripheral processes from the different sensory modalities terminate within a fixed temporal interval, and that the amount of crossmodal interaction manifests itself in an increase or decrease of second stage processing time. A qualitative test is consistent with the model prediction that the probability of interaction, but not the amount of crossmodal interaction, depends on target–nontarget onset asynchrony. A quantitative model fit yields estimates of individual participants' parameters, including the size of the time window. Some consequences for the design of driver assistance systems are discussed.

## Introduction

The capacity of humans to simultaneously process information from separate sources is inherently limited see[1], [2]. This limitation is particularly conspicuous in a traffic situation: the act of driving is a highly complex skill requiring the sustained monitoring of perceptual - predominantly visual - and cognitive inputs [3], [4]. A driver has to constantly monitor both the state of the vehicle and the behavior of other traffic participants. The limited capacity of humans to divide their attention amongst all of the competing sensory inputs [5] is further challenged by the introduction of modern in-vehicle devices like cell phones or navigation systems. Recent developments of driver assistance systems, like front-collision warning or lane-change assistance systems, are aimed at alleviating the human workload. However, some of these systems present their information on the windshield using of visual overlays (“head-up display” technologies) presenting yet another source of information to be processed by the driver.

In order to ease visual information overload, the design of complex human-machine interfaces like driver assistance systems has shifted towards utilizing additional, non-visual perceptual channels with auditory and tactile stimulation devices, in particular. For example, several automotive vendors offer a lane departure warning system, which uses haptic feedback in form of a vibration on the steering wheel to inform the driver that she is about to leave her current driving lane. Future cars with a head-up display (that can overlay visual items on the windshield) might combine the tactile stimulus with a specific visual warning. An auditory or tactile signal reduces the rate of visual information to be processed at a given point in time, but it also entails the potential occurrence of multisensory integration effects. Such effects are mostly facilitatory: typically, human orienting responses towards an audiovisual warning signal tend to be faster and more reliable than to a unimodal signal. However, crossmodal signals may also cause inhibitory effects, that is, a slowed response or increased error rate.

Importantly, certain rules specifying the spatiotemporal arrangement and signal intensity levels of the unimodal components necessary and/or sufficient for the occurrence of multisensory integration have been formulated [6]. First, stimuli from different modalities must be in close temporal proximity in order for multisensory integration to occur at all (*temporal rule*). Second, the effects are the larger the closer in space the stimuli are presented (*spatial rule*). Third, the magnitude of the multisensory effect is inversely related to the strength of the stimuli presented (principle of *inverse effectiveness*).

The question addressed here is whether, and to which degree, these multisensory integration rules, so far mostly observed under more confined laboratory environments, are still valid in a less controlled environment like in a driving simulator. Obviously, taking these rules into account is of utmost importance in designing optimal crossmodal human-machine interfaces like driver assistance systems. Note that, although the experiment reported here took place in a driving simulator setup, our primary goal was not to create a realistic driving situation, e.g., involving interaction with the participants' vehicle or other road users. Rather, we focus on how the effectiveness of designing crossmodal configurations for improving orienting attention towards icons delivered by the assistance system is constrained by the wellknown multisensory integration rules. Moreover, it will be probed whether the observed results are consistent with the ‘time window of integration’ (TWIN) modeling framework that is introduced below.

An established method of probing multisensory integration rules is to measure the speed of saccadic reaction time (SRT), i.e., the time from the presentation of a target signal to the beginning of the eye movement towards the target position [7]. In many studies, SRTs to visual targets have been shown to be affected by the presence of auditory or tactile non-targets presented in spatiotemporal proximity of the target [8]–[14]. Generally, mean SRT to a visual target is reduced by a spatially coincident auditory non-target (between 10 to 50 ms), the effect decreases monotonically with increasing spatial distance, although sometimes an inhibitory effect for large distances has been found as well. Moreover, the effect of spatial distance is modulated by the level of background noise in which the auditory distracter is embedded [15], [16].

The temporal rule of multisensory integration has been instantiated via the concept of a *time window of integration* [17]. It refers to a temporal interval within which stimuli of different modalities must be registered by the perceiver for an intersensory effect to occur. Although a *window of integration* has originally been defined for both spatial and temporal aspects of a crossmodal experiment [18] and has even been suggested for higher-level aspects like semantic congruity [19], we will confine discussion to the temporal dimension within the reaction time context considered here. Based on this concept, Colonius and Diederich [20] have developed the time-window-of-integration (TWIN) model for saccadic reaction times. It is a quantitative framework that predicts the effect of the spatiotemporal parameters of a crossmodal experiment on response speed. The TWIN model postulates that a crossmodal stimulus triggers a race mechanism in the very early, peripheral sensory pathways which is then followed by a compound stage of converging sub-processes comprising neural integration of the input and preparation of a response. Note that this second stage is defined by default: it includes all subsequent, possibly temporally overlapping processes that are not part of the peripheral processes in the first stage. The central assumption of the model concerns the temporal configuration needed for crossmodal interaction to occur: *Crossmodal interaction occurs only if the peripheral processes of the first stage all terminate within a given temporal interval*, the ‘time window of integration’ (TWIN assumption). Thus, the window acts as a filter determining whether afferent information delivered from different sensory organs is registered close enough in time to trigger multisensory integration. Passing the filter is necessary, but not sufficient, for crossmodal interaction to occur because the amount of interaction may also depend on many other aspects of the stimulus set, like the spatial configuration of the stimuli. The amount of crossmodal interaction manifests itself in an increase or decrease of second stage processing time, but it is assumed not to depend on the stimulus onset asynchrony (SOA) of the stimuli. A formal presentation of the model is given in the methods section below.

Although the TWIN model's assumptions certainly oversimplify matters, they afford quite a number of experimentally testable predictions, many of which have found empirical support in recent studies cf. [16], [21]–[24]. For the *focused attention paradigm* (FAP) used here, the model is further specified by one important assumption: *Crossmodal interaction occurs only if (i) a nontarget stimulus wins the race in the first stage, opening the time window of integration such that (ii) the termination of the target peripheral process falls in the window*. One interpretation is that the winning non-target will keep the system in a state of heightened reactivity such that the upcoming target stimulus, if it falls into the time window, will trigger crossmodal interaction. For saccadic eye movements, in particular, this may correspond to a gradual inhibition of fixation neurons (in superior colliculus) and/or omnipause neurons (in midline pontine brain stem). If a stimulus from the target modality is the winner of the race in the peripheral channels, second stage processing is initiated without any multisensory integration mechanism being involved. A more detailed quantitative description of the model is found in the materials and methods section.

**Figure 1:**
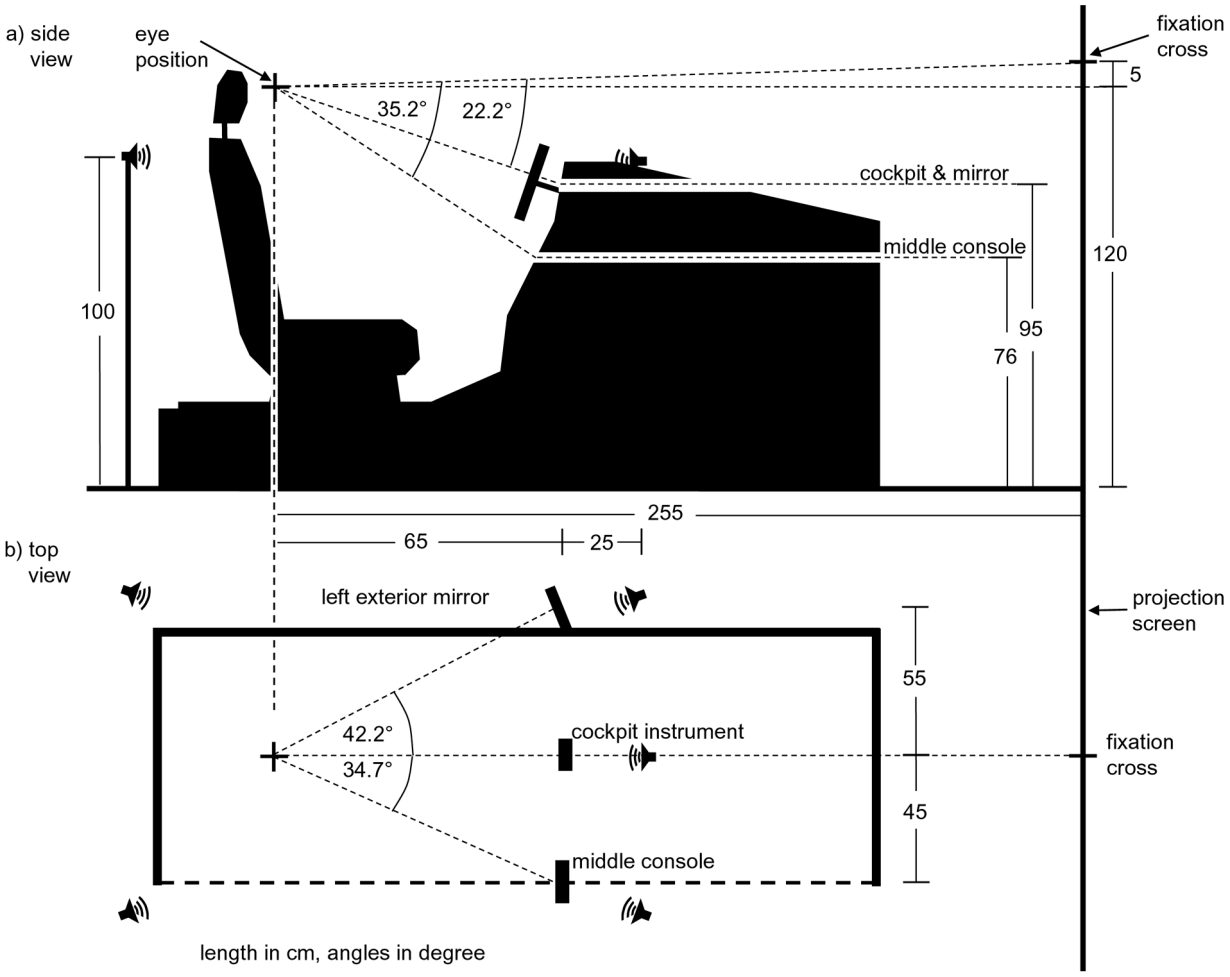
Fixed-based driving simulator. Illustration of the driving scene including fixation cross and visual targets presented to the participants: in the left outside mirror the red border cue is depicted, in the cockpit display the white arrow cue.

The present experiment was conducted in a driving simulator with a front beamer (field of view is 60 degrees) presenting an autobahn scenario of a steady flow scene. The assistance system consisted of a blind spot detection system (a red visual icon in the corresponding outside mirror) and a lane change assistant (a white arrow in the cockpit) that served as visual targets (for details, see below and Figure 1). The focus here is on how much the saccadic response time of participants, i.e., the time to start moving the eyes toward a visual signal provided by the assistance system, is speeded up by presenting a distractor stimulus (also called non-target or accessory), here a vibration on the steering wheel or an acoustic signal. The time to respond to signals presented by the assistance system is obviously an important aspect of a traffic situation studied previously. For example, [25] investigated a rear-end collision warning system that signalizes the driver a rapidly slowing-down lead car (with/without defect braking lights) and found that a multimodal audio-tactile warning signal reduced reaction time by 10 percent compared with the presentation of unimodal auditory warning signals alone. Moreover, compared to unimodal stimuli, a peripheral multisensory stimulus is able to cue attention to a specific location even under high perceptual load [26].

## Materials and Methods

### Ethics Statement

Participants gave their written informed consent prior to their inclusion in the study and the experiment has been conducted according to the principles expressed in the Declaration of Helsinki. Approval for this study was granted by the Academic Integrity Committee of Oldenburg University.

### Equipment and stimulus presentation

**Figure 2:**
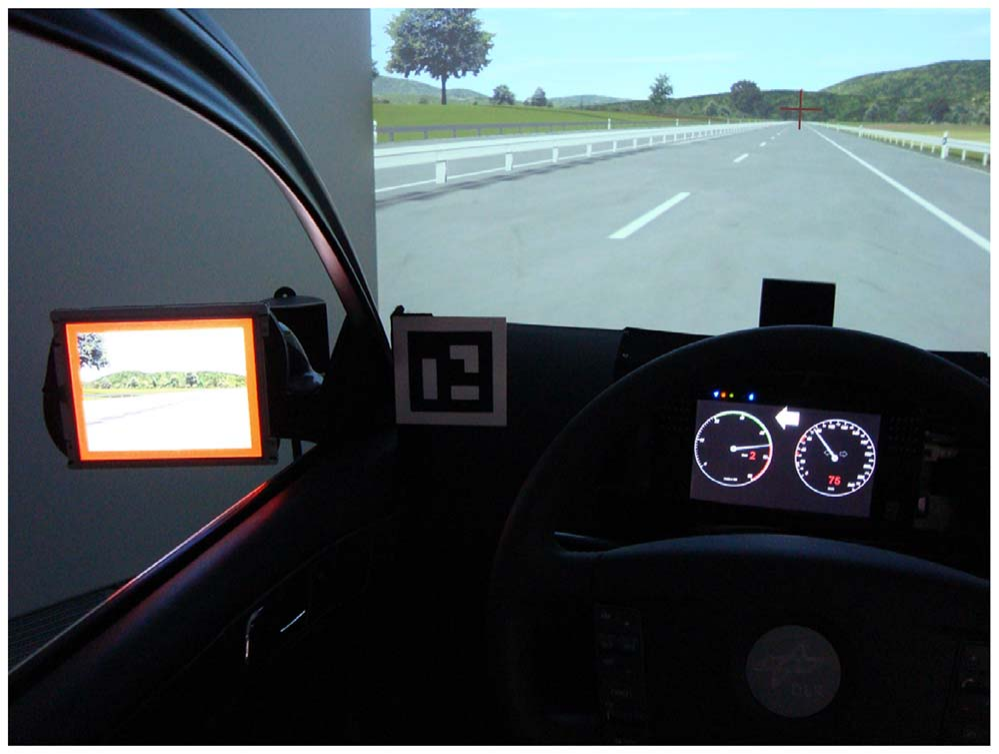
Dimensions of the driving simulator. a) Schematic side view of the simulator. b) Birds-eye view. Rear speakers and middle console elements were not used in this experiment.

The experiment was performed in a fixed-based driving simulator (see Figure 2) with a front beamer (Canon XEED SX6) and a field of view of 60 degrees horizontally and 40 degrees vertically (1400 by 1050 pixels). The visual stimuli were presented in the left outside mirror (17 by 13 cm, with a resolution of 1024 by 768 pixels) and the cockpit instrument (15 by 9 cm, 1024 by 600 pixels). The cockpit instrument presented a white arrow on black background used for navigational hints in an assistance system, the cue in the mirror was a red border around the entire mirror used as blind spot warning about an approaching car. The left and right speakers (front and rear) were each mounted on a pole, the center speaker was placed on the simulator itself. Background engine noise was presented via the center speaker (43 dB SPL) and the directional acoustic cues were presented via the center or left front speaker. Those sound stimuli were (i) white noise (48 dB SPL, sampling rate 44,100 Hz) and (ii) a specific alarm sound used by several assistance system functions installed in our simulator. For the presentation of acoustic stimuli the simulator uses OpenAL software (http://connect.creativelabs.com/openal/default.aspx) for Dolby 5.1 simulation which allows precise positioning of stimuli.

The simulator comes with active pedals and steering wheel using Schneider Lexium LXM05 CAN Bus servo drives for haptic feedback. The haptic vibration cue applied on the steering wheel was a sinusoidal wave with a frequency of 50 Hz. The 220 Volt drive is controlled via so-called “current control” mode and has a power range of 0–27.9 Ampere, controlled by a discrete signal range from 0–32767. Due to this control mode no force in Nm is applied directly. We tried to measure the force with a spring scale but this did not work very well, hence we can not report an exact force value for the vibration. The driving simulator software used in this experiment was SILAB (www.wivw.de). It was extended with a module to trigger the stimuli including a data recording unit. The module was plugged into the simulator with at a sampling rate of 120 Hz. With this setup, signal processing and recording were synchronized. To measure reaction times the head mounted dikablis eye tracker system was used (www.ergoneers.de).

### Data recording and preprocessing

The dikablis eye tracker system has 25 Hz sampling rate (40 ms). Clock synchronization of simulator and eye tracker was done each time at simulation start and both systems created a continuous file output recording. Both recordings (stimulus recording and eye data) were merged for data analysis purpose. No interpolation was used, missing values due to asynchronous time stamps were filled with the latest available values. Because of the rather low sampling rate of the eye tracker (40 ms) the full time for the eye movement is recorded within a couple of samples. Furthermore, the system is based on an video-based approach that records an eye and a scene video file. The resulting data file contains the coordinate information of the gaze direction projected in the scene video. With the known distance between head of the participant and the projection screen the angular movement can be calculated.

The driving simulator as well as the eye tracker separately record their data into comma separated files. They continuously write streams with 120 Hz (simulator) and 25 Hz (eye tracker). The simulator contained the unit for experiment control, which triggers the signals for each trial, thus the file contains a time stamp and several columns for each possible signal which are 0 if the signal is not present or 1 if the signal is present. The eye tracker uses an infrared camera with a resolution of 384 by 384 pixels and records a coordinate pair each time the eye could be tracked successfully, or zero if tracking was not possible.

To synchronize both units, the simulator was used as master who initially resets the time stamps on both units. A differential comparison of those recorded time stamps showed slight wavelike displacement over the time (+−16 ms), but no increasing bias between the units. The process for merging these two files is shown in Table 1.

**Table 1:** Merging two asynchronously recorded data files (Example).

Simulator Data		Eye Tracker Data		Merged Result		
Timestamp	SimData	TimeStamp	EyeData	Timestamp	SimData	EyeData
…	…	0	10	0	0	10
24	0	40	20	24	0	10
32	1	80	30	32	1	10
40	2			40	2	20
48	3			48	3	20

Data of the driving simulator (left), the eye tracker (middle) and how the data is merged (right). No interpolation, the latest valid value is taken to fill in missing time slots.

### Participants

Seven students (4 female), aged 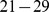, served as paid voluntary participants. All had normal or corrected-to-normal vision, and were naive as for the purpose of the study. They were screened for their ability to follow the experimental instructions (proper fixation, few blinks during trial, saccades towards visual target). They gave their informed consent prior to inclusion in the study. Participants were allowed to quit the experiment at any time.

### Procedure

Before calibration started, participants were instructed to take a comfortable driving seat position, to put the head against the headrest (in order to reduce movement during data recording), and to keep both hands on the steering wheel. Participants became dark adapted while the eye movement registration system was adjusted and calibrated. Their task was not to drive actively; instead, the computer controlled the ego-car's movement. A straight two-lane autobahn appeared on the screen to create a realistic visual impression including the optical flow. The scene consisted of a straight country road with some typical visual decorations (reflector posts, trees). No further traffic was shown. The ego-car accelerated automatically up to 120 km/h.

**Figure 3:**
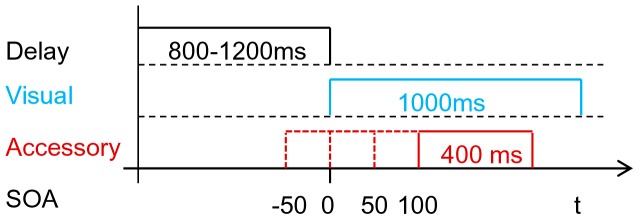
Timecourse of a trial. At each trial a visual (target) stimulus of 1000 ms duration was presented after a time period of 800–1200 ms (uniform distribution range). Onset of accessories (acoustical or tactile stimuli of 400 ms duration) occurred at 4 different levels of stimulus onset asynchrony (SOA) relative to the target.

In the middle of the screen a fixation cross was continuously present. After 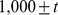 ms, with 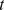 drawn from a uniform distribution with range 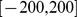, a visual target was presented, either in the cockpit of the simulator (a white arrow showing to the left) or in the left outside mirror (a red border), with equal probability(see Figure 1). Participants were instructed to gaze at the visual target as quickly and accurately as possible ignoring any other stimulus (focused attention paradigm). When the visual stimulus was extinguished, they had to turn back their gaze to the fixation cross. Trials lasted for 4.5 s. Depending on the particular condition, the visual target appeared alone, or with an auditory accessory (a white noise or a beep), or a tactile accessory (vibration on the steering wheel). The onset of the accessories was shifted relative to the visual targets (*stimulus onset asnychrony*, SOA) by 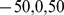, or 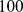 ms (negative SOA values: accessory presented prior to the target). The visual targets were presented for 1,000 ms, the accessories for 400 ms (see Figure 3). Relative spatial position was also varied (coincident or disparate configuration). Additionally, catch trials (auditory or tactile) were presented. Here, participants' task was not to react at all and to keep gaze on the fixation cross. Because the tactile stimulus was only administered to the steering wheel, visual-tactile configurations were defined as (i) *coincident*: white arrow in the cockpit and (ii) *disparate*: red border in the outside mirror.

### Presentation schedule and error detection

Each block of presentations contained 87 trials. The first 2 blocks were used for training and did not enter the results. One session contained four blocks and participants were allowed to make a short break after two blocks. Presentation order in each block was completely randomized over conditions. Each block lasted about 10 min. Four of the seven participants completed 46 blocks, two of the participants 44 blocks, and one participant 38 blocks, resulting in a total between 3,306 and 4,002 trials, depending on subject availability. Data collection was spread over a period of 2 weeks.

Trials where the eye tracker recorded ‘invalid’ values during stimulus presentation, i.e., where the eye could not be tracked, were eliminated (see Table 2). The remaining data were screened for three different types of errors (see Table 3): (i) anticipation (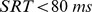), (ii) misses (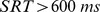), and (iii) direction (gaze moved towards the wrong display). For the removal of misdirected gazes we calculated the angle 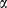 between the two vectors 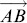 and 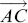, with A = fixation cross, B = mirror, C = left mirror. A trial was labeled as *misdirection* if the saccade deviated from the target direction by more than 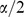.

**Table 2:** Total number of trials per subject (87 trials per block).

Subject	Total Trials (Blocks)	Invalid
VP1	4002 (46)	1664
VP2	4002 (46)	1580
VP3	3654 (42)	744
VP4	3654 (42)	520
VP5	4002 (46)	419
VP6	4002 (46)	195
VP7	3306 (38)	102

Number of blocks varied due to availability of subjects. *Invalid*: pupil not detected correctly during trial.

**Table 3:** Total number of trials per subject that could be used for data analysis; three categories of error were filtered: (1) anticipation errors: 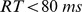, (2) misses: 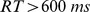, (3) misdirected: visual target cue on left mirror, but initial gaze response direction was towards cockpit or vice versa.

Subject	Total	Errors
VP1	2338	8
VP2	2422	23
VP3	2910	21
VP4	3134	53
VP5	3583	79
VP6	3807	2
VP7	3204	41

### Time Window of Integration (TWIN) Model: Details

The race in the first, peripheral stage of the model is made explicit by postulating nonnegative, statistically independent random variables for the processing times of (i) visual targets, 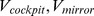, (ii) auditory nontargets, 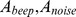, and (iii) the tactile nontarget (vibration), 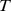. For crossmodal interaction to occur, a nontarget stimulus must win the race in the first stage and the target peripheral process must terminate before the time window is closed. Thus, writing 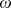 for the width of the integration window and 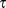 for the specific SOA value, the condition for interaction is the event 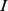 to occur,
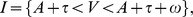
where 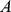 stands for one of the accessory (nontarget) stimulus processing times 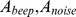 or 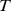, and 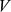 stands for one of the target stimulus processing times, 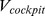 or 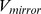. Thus, the probability of integration to occur, 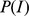, is a function of both 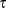 and 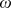; it can be determined numerically once the probability distribution functions of 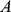 and 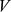 have been specified. For the present experiment, the model allows for different 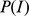 functions for each pairing of a target (cockpit, mirror) with an accessory nontarget (beep, noise, vibration).

The next step is to compute expected reaction time for the unimodal and crossmodal conditions. From the two-stage assumption, total reaction time in the crossmodal condition (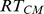) can be written as a sum of two random variables:
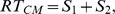
where 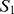 and 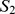 refer to the first and second stage processing time, respectively. In the FAP version of TWIN considered here, first stage processing terminates with the target peripheral process, thus 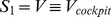 or 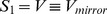, depending on the identity of the target. For the expected saccadic reaction time in the crossmodal condition then follows:
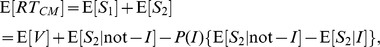
where 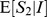 and 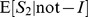 denote the expected second stage processing time conditioned on interaction occurring (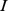) or not occurring (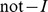), respectively. Setting
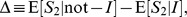
this becomes
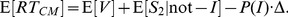
(1)In the unimodal condition, no integration is possible. Thus, for unimodal response time 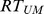,
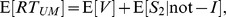
and we arrive at the simple product rule for expected crossmodal interaction (ECI)
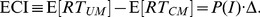
(2)In the present experiment, the model allows for a value of 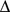 for each pairing of a target (cockpit, mirror) with an accessory nontarget (beep, noise, vibration) in both the coincident and the disparate condition, for total number of 12. According to TWIN, Equation 2 expresses the separation of temporal and non-temporal factors for the observable ECI : the first factor, 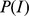, depends on SOA and target/nontarget intensity parameters, whereas the second factor, 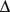, depends on crossmodal properties, like spatial separation, but not on SOA or intensity parameters.

Before we turn to a parametric version of TWIN, we consider testing the model without making specific assumptions about the distribution of the component processing times ([22], [23]). It is obvious from the product rule that, whenever TWIN predicts that two experimental conditions differ either with respect to 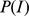 or 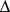 but not to both, the corresponding ratio of ECIs should no longer depend on the parameters of the cancelled terms. For example, in the ratio of two ECIs that differ with respect to the spatial condition (coincident vs. disparate) the 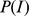 terms cancel for a given SOA value. In other words, this ratio should be invariant, within statistical error variability, across SOAs. This prediction will be tested below.

For the parametric TWIN version, in order to numerically estimate the parameters 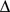 and 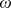 one has to introduce specific distributions for first and second stage processing times. While many –more or less arbitrary– options for these distributions exist, an exponential-Gaussian model specification has been probed in a number of our empirical studies ([16], [24]). It assumes statistically independent exponential random variables for all first stage processing times, each with a specific parameter 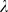, plus a Gaussian distribution for second stage processing time with mean 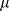 and standard deviation 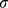. The ex-Gaussian expressions for 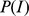 and for the expected uni- and crossmodal response times are found in the Appendix at the end of this article.

## Results

### Statistical analyses on SRTs

**Figure 4:**
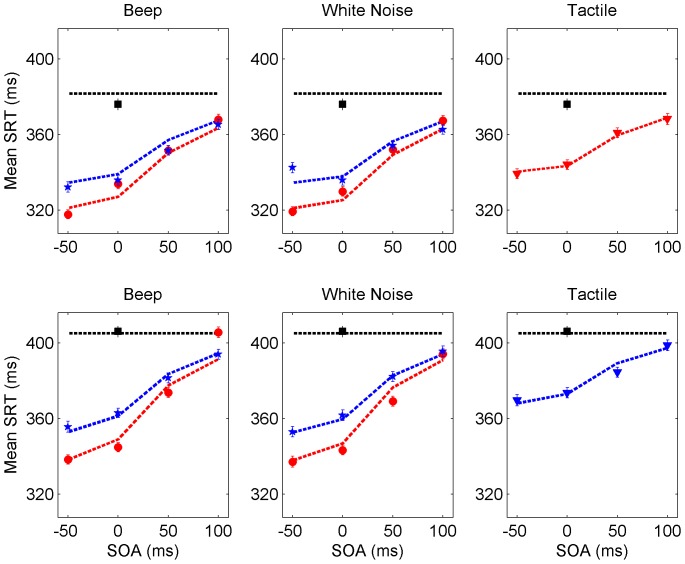
Results across all seven participants. Observed (symbols) and predicted (curves) mean SRTs as a function of SOA for coincident (red) and disparate (blue) stimuli for cockpit (upper panels) and mirror conditions (lower panels). Note that in the tactile conditions only one spatial configuration for each visual target was presented (for the cockpit condition coincident and for the mirror condition disparate, respectively). Black colour indicate unimodal visual mean SRTs.

On average, participants responded faster to the visual targets when they were accompanied by an auditory accessory (t-test (two-tailed) comparing the visual alone condition (390 ms) with the audio-visual condition (356 ms): t = 46.63, 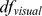 = 963, 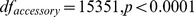). A within-subjects analysis of variance (ANOVA) was performed on SRTs with fixed factors *auditory nontarget* (white noise vs. beep), *spatial configuration* (coincident vs. disparate), and *stimulus onset asnychrony (SOA)* (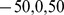, and 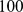 ms), and *participants* as random factor. There were main effects of *spatial configuration* 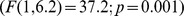 and *SOA* 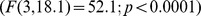 but not of *auditory nontarget* (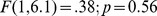). There was a two-way interaction between *SOA* and *spatial configuration* 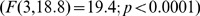 indicating that the difference in SRT between the coincident and disparate condition diminishes over increasing SOA (see Figure 4).

Random factor *participants* was significant suggesting interindividual differences in the general speedup of SRT 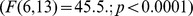. Moreover, there was a significant interaction between *participants* and *SOA* 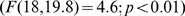 pointing to individual differences in the SRT-SOA curves. From visual inspection of the individual panels three participants (P3, P6, P7) show a dip in the SRT-SOA curve at SOA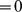 in two of their 6 conditions. Finally, there were individual differences among participants concerning the interactions between *auditory nontarget* and *SOA* (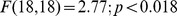) and *auditory nontarget* and *spatial configuration* (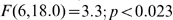)

ANOVAs for SRTs with the tactile accessories, separately for mirror condition and cockpit condition, were performed with *SOA* as fixed factor and *participants* as random factor. For both there was a significant effect of *SOA* (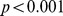). As before, the speedup diminishes with delaying the accessory and there were interindividual differences in the SRT-SOA curves (interaction *SOA* and *participant* 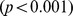). However, the amount of facilitation was the same with the cockpit or the outside mirror target considering that the absolute average SRT is lower to the former (379 ms) compared with the latter (406 ms) condition (t-test (two-tailed): 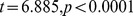).

### Main spatiotemporal effects in SRT

Mean saccadic reaction times (± standard error) across all participants are plotted in Figure 4, and Figures S1, S2, S3, S4, S5, S6, S7 depict the data separately for each participant (curves representing model predictions). Although there are individual differences in overall speed, the participants' response patterns across conditions are quite similar:

First, reaction time to visual targets is facilitated in the presence of auditory or tactile nontargets, and the effect tends to be the larger the earlier the nontarget is presented, within the limited set of SOA values. Second, the reaction time speedup is larger when target and auditory nontarget are spatially coincident compared to the spatially disparate configuration, making both findings consistent with the ubiquitous temporal and spatial rules of multisensory integration described in the introduction.

Third, whether beep or white noise was accompanying the visual target did not have much of an effect on the speedup of saccadic reaction times; this is possibly due to the similarity of the nontargets with respect to intensity level and localizability. Fourth, the speedup is less pronounced with the tactile (vibration) nontarget than with the auditory nontargets, especially when vibration occurs 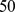 ms before or simultaneously with the target. It cannot be ruled out, though, that presenting the tactile stimulus even earlier than 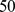 ms would achieve a speedup comparable to the one with auditory nontargets. Finally, there was no spatial effect with the tactile nontarget, that is, it did not matter whether the accessory vibration was paired with the cockpit or the outside mirror target. This lack of an effect may not be surprising given that the source of vibration, the steering wheel, was not close to the cockpit target nor the outside mirror target.

### Fit of TWIN model

#### Qualitative test

This test is based on the product rule for expected crossmodal interaction (ECI) from Equation (2),
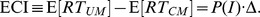
We assume two different values of the amount of interaction for coincident and for disparate presentation of target and nontarget, 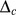 and 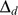 and consider the ratio of the corresponding ECIs,
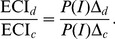
(3)According to the model assumptions, the probability of interaction 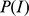 depends on the intensity parameters (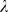) of the first-stage processing times, on the SOA value, and on time window size (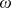) but not on the interaction parameters (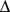). Therefore, 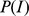 cancels and the ratio of ECIs should not depend on the specific SOA value. Because there was no significant difference for the beep and the white noise condition, this ratio should also be invariant with respect to a change of the auditory accessory. Table 4 lists the value of the ECI ratios for each of the 8 conditions (4 SOAs, beep/white noise) for all participants. The null hypothesis of no difference in these 8 conditions was tested by the nonparametric Friedman rank sum test. No statistically significant deviation was found (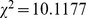, df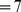, p-value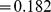).

**Table 4:** Estimates of ECI ratio for *disparate/coincident* conditions.

Participant	Beep with SOA [ms]				White noise with SOA [ms]			
	−50	0	50	100	−50	0	50	100
1	0.7778	0.8676	0.7959	0.6571	0.7188	0.8148	0.8448	1.1111
2	0.6786	0.8537	0.4667	−3.000	0.6897	0.5111	1.2222	1.5000
3	0.8667	1.0270	1.6667	−1.833	0.7333	0.7568	0.6250	−0.166
4	0.7206	0.9778	1.0385	1.3750	0.6471	1.0000	1.0000	4.3333
5	1.0805	0.9474	0.8667	1.8889	0.7500	1.1967	1.0741	1.7692
6	0.3429	1.0667	2.1429	0.7500	0.0571	0.9062	0.7500	−0.3333
7	0.5897	0.8276	1.2222	1.4000	0.3095	0.8235	1.0500	1.0833

#### Fit of ex-Gaussian TWIN model

For each target and nontarget, one parameter (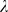) for the exponential peripheral processing time has to be estimated. For second stage processing, we estimate separate mean values for the cockpit and the outside mirror target, 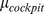 and 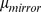, given no interaction occurs. Since the ANOVA did not find a main effect of auditory accessories, the interaction parameters for beep and white noise were set equal in the estimation procedure, i.e., there are only two visual-auditory 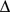 values, one for coincident and one for disparate presentation of the auditory nontargets, denoted 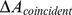 and 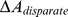. There are also two interaction parameters to be estimated for the tactile nontarget conditions, cockpit mirror and outside mirror, denoted as 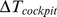 and 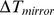. Since the values for SOA (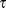) are given, the final parameter to be estimated is the width of the integration window, 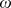. In order to avoid the estimation routine to run into implausible parameter estimates, certain restrictions for all parameter were imposed a-priori.

The left column of Table 5 lists all parameters of the TWIN model to be estimated, while the middle column shows the parameter restrictions that were imposed on the estimation routine. The 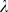 values were restricted to fall within a range consistent with neurophysiological estimates for peripheral processing times ([6], [27]). The width of the the time window of integration, 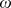 was limited to a lower bound of 150 ms. For simplicity, the same 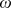 was assumed for auditory and tactile nontargets (but see [23]). These 12 parameters were estimated separately for each participant from 42 means (40 bimodal, 2 unimodal) from all valid observations.

**Table 5:** Restrictions to model parameters in the estimation routine.

Parameter	Restriction Limits (in ms)	Mean central/peripheral processing time
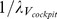	20–200	peripheral, for visual target inside cockpit
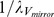	20–200	peripheral, for visual target in outside mirror
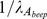	20–200	peripheral, for auditory nontarget, beep
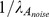	20–200	peripheral, for auditory nontarget, noise
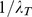	20–200	peripheral, for tactile nontarget
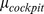	>0	central, for visual stimuli in cockpit
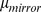	>0	central, for visula stimuli in mirror
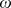	≥150	window of integration
		amount of crossmodal interaction due to
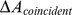	none	auditory stimuli presented coincident
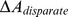	none	auditory stimuli presented disparate
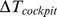	none	tactile stimulus presented coincidental
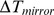	none	tactile stimulus presented disparate

Parameter estimation was performed by minimizing the Pearson 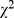 statistic over observed and predicted mean SRT using the FMINSEARCH routine of MATLAB:
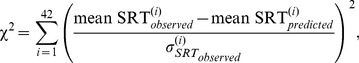
(4)with 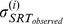 denoting the standard error for each of the 42 conditions.

Table 6 lists all resulting parameter estimates separately for each participant, the final column presents estimates for the combined data. The last row lists the goodness-of-fit 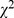 values for each participant.

**Table 6:** Parameter estimates for TWIN model.

				Participant				
Parameter	1	2	3	4	5	6	7	all
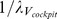	121	60	96	96	81	114	134	90
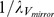	87	50	95	70	78	94	63	71
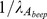	36	26	20	29	31	24	25	23
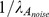	20	21	48	26	28	20	20	20
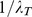	20	51	34	20	66	20	20	20
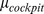	297	376	273	315	311	217	213	291
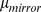	366	384	297	347	330	271	340	335
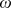	182	150	150	150	223	150	150	150
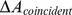	131	77	77	106	100	72	69	85
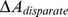	111	62	69	84	90	50	47	66
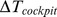	114	64	54	44	92	50	47	58
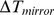	83	35	56	48	99	30	31	47
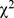	46.0	32.2	88.9	40.9	31.0	88.6	68.5	93.6

Figure 4 plots observed mean SRT values (± standard error) against model predictions across SOAs for all experimental conditions averaged over participants. Individual data fits are plotted in Figures S1, S2, S3, S4, S5, S6, S7. In the upper row panels, the visual target stimulus was presented in the cockpit mirror; in the lower row panels, the target was presented in the outside mirror of the car. Circles (resp., asterisks) refer to mean SRTs to bimodal stimuli presented coincident (resp., disparate), and the corresponding model predictions are presented by dashed lines (in the same color as the data points). The horizontal dashed line indicates the estimated mean unimodal (visual) SRT.

Figure 4 suggests that the TWIN model captures the aggregated (over participants) data quite well for all experimental conditions. This is also true on the level of individual participants except for a number of specific but unsystematic deviations at some data points. Considering a formal goodness-of-fit criterion, however, only participants 2, 4, and 5 have 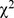 value that are not significant at the 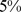 level (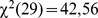) where 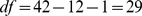.

## Discussion and Conclusion

Modern driving assistance systems make increasingly use of auditory and tactile signals in order to reduce the driver's visual information load (see, e.g. [4]). For the design of these non-visual signals to be most effective, either in drawing attention to a source of visual information or in providing relevant information by themselves, certain effects of crossmodal sensory interaction should, arguably, be taken into consideration. Here we have shown that indeed some well-known spatiotemporal rules of multisensory integration, usually found under confined laboratory conditions (but see [14] for a study with a complex audiovisual scene) also apply to results obtained in a driving simulator environment that was located in an “ordinary” office without sound attenuation or complete darkness.

Many multisensory studies with more complex tasks involving visual discrimination and driving-specific motor actions have already been conducted and will continue to play an important role in human-machine interface design [28]–[34]. Here, in order to make our results comparable to those gained in more controlled laboratory studies, our participants' task was restricted to simple eye movements towards well-defined driving-specific targets, i.e., outside mirror and cockpit displays. Specifically, we have found that auditory and tactile accessory stimuli can reduce saccadic reaction time up to 80 ms depending on the spatiotemporal configuration. For auditory stimuli, the speedup is most pronounced when visual target and auditory nontarget were spatially coincident (same direction in space) and the auditory was presented 50 ms prior to the visual [8], [15], whereas for the tactile stimulus (vibration of steering wheel) the speedup was typically not more than 40 ms and no spatial effect was found. This is consistent with the findings of [34] that the effect of a tactile stimulus is only effective in capturing spatial attention when combined with an auditory stimulus presented from the same direction. However, since here no combined audio-tactile stimuli were presented it remains an open question whether such a spatial effect would have been observed in our driving simulator as well. It should be mentioned that overall the spatial effect is only about 20 ms, somewhat smaller than what can usually be found in a laboratory setting [8], [21]. This may be due to participants' limited spatial hearing in the car environment, which may also explain why no significant difference on SRT was found for the white noise vs the beep stimulus.

The facilitatory effect of the accessory stimulus on SRT was shown to decrease the later the auditory or tactile stimulus was presented relative to target onset, within the limited range of SOAs from 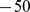 ms (before the visual target) up to 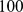 ms (after the visual target) employed in this study. This is in agreement with the “temporal rule” found in numerous laboratory studies postulating temporal proximity as a condition for multisensory integration to occur. In a recent study by Ho, Spence, and colleagues [35], even exact synchronous presentation of stimuli was required for maximum facilitation in a head-turning audiovisual orienting task.

In any event, our results revealed that the temporal mechanism of the crossmodal effects in the driving simulator can be described appropriately with the TWIN model [20]. Given that stimulation in the driving simulator was much more complex and the physical environment much less controlled than in previous laboratory experiments, the good performance of the model is surprising. We do not claim that the model renders a correct representation of the underlying processes in each and every detail, in particular given that model fitting was restricted to mean (average) data whereas recent modeling efforts suggest that the level of response variability may be an important component of multisensory integration [36]. Still, as demonstrated by the parameter-free test using ECI ratios, the basic assumptions of the model are being corroborated. Obviously, individual participants' data vary in their goodness of fit to the model and due to the nontrivial parameter estimation task the exact parameter values need to be taken with a grain of salt. Nevertheless, the pattern of parameter values yields some potentially informative insights. For example, we found that across conditions reaction time to the cockpit stimulus was faster than to the mirror stimulus. This might have been attributed to the difference in physical properties of the target type (red border around mirror vs. white arrow on dark background) but, surprisingly, the parameters suggest that peripheral processing for the cockpit stimulus took more time than for the mirror stimulus in all participants, whereas the opposite holds for processing time in the more central stages. One possible explanation –that will need further scrutiny– is that the temporal resolution might be higher in the horizontal than in the vertical plane of the retina and is hence more sensitive for detecting changes in the environment (first stage of the TWIN-model). On the other hand moving gaze from the fixation point on the screen to the mirror stimulus could involve a more time consuming motor programming process because eye movement in two dimensions (vertical and horizontal) must be activated contrary to the cockpit condition where only one dimension (vertical) is addressed (second stage of the TWIN-model).

Whether or not the facilitatory effect of the nontargets is due to “true” multisensory integration or a certain type of “warning effect” has been subject to some controversy. As discussed in [37], the alternative to integration is the notion that “a salient but spatially non-predictive cue event in one sensory modality may attract multisensory covert spatial attention to its location and might do so automatically, even when the cue modality is task-irrelevant.”(ibd., p. 308). Whereas this issue is of interest from a theoretical point of view, it has no direct impact on the conclusions about human-machine interface design of this study.

The foremost conclusions to be drawn from the current findings is that (i) the multisensory spatiotemporal integration rules found in laboratory studies need to be considered in designing efficient driving assistance systems, and that (ii) the TWIN model is a useful tool in describing and predicting these crossmodal effects in detail. The observed speedup of responses produced by nontarget auditory and tactile stimulation in the order of 80 ms may at first sight not appear to be significant, but one should keep in mind that in a complex traffic situation timing can be rather critical and that several of these effects may combine additively.

## Appendix

### TWIN model: Ex-Gaussian version

#### Probability of integration 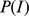


The peripheral processing times 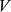 for the visual and 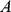 for the visual stimulus have an exponential distribution with parameters 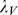 and 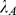, respectively. That is,
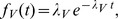


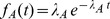
for 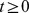, and 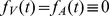 for 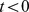. The corresponding distribution functions are referred to as 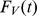 and 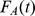.

The visual stimulus is the target and the auditory stimulus is the nontarget. By definition,
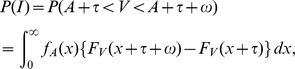
where 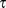 denotes the SOA value and 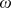 is the width of the integration window. Computing the integral expression requires that we distinguish between three cases for the sign of 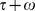:

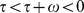

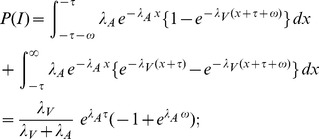

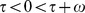

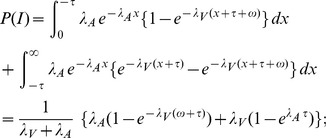

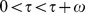

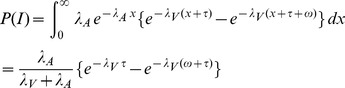

#### Mean reaction times

The mean RT for crossmodal stimuli is
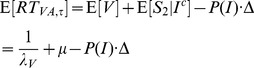
and the mean RT for the visual target is
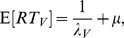
where 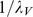, the mean of the exponential distribution, is the mean RT of the first stage and 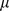 is the mean RT of the second stage when no interaction occurs.

### Participant data

Figures S1, S2, S3, S4, S5, S6, S7 show the results for all seven participants seperately. Observed (symbols) and predicted (curves) mean SRTs as a function of SOA for coincident (red) and disparate (blue) stimuli for cockpit (upper panels) and mirror conditions (lower panels). Note that in the tactile conditions only one spatial configuration for each visual target was presented (for the cockpit condition coincident and for the mirror condition disparate, respectively). Black colour indicate unimodal mean SRTs.

## Supporting Information

Figure S1 **Observed and predicted data for VP1.**
(TIF)

Figure S2 **Observed and predicted data for VP2.**
(TIF)

Figure S3 **Observed and predicted data for VP3.**
(TIF)

Figure S4 **Observed and predicted data for VP4.**
(TIF)

Figure S5 **Observed and predicted data for VP5.**
(TIF)

Figure S6 **Observed and predicted data for VP6.**
(TIF)

Figure S7 **Observed and predicted data for VP7.**
(TIF)
