# Supplementary figures and images for: Designing Driver Assistance Systems with Crossmodal Signals: Multisensory Integration Rules for Saccadic Reaction Times Apply
Source: PLoS One. 2014 May 6;9(5):e92666. doi: 10.1371/journal.pone.0092666 (PMC4011748; doi:10.1371/journal.pone.0092666)

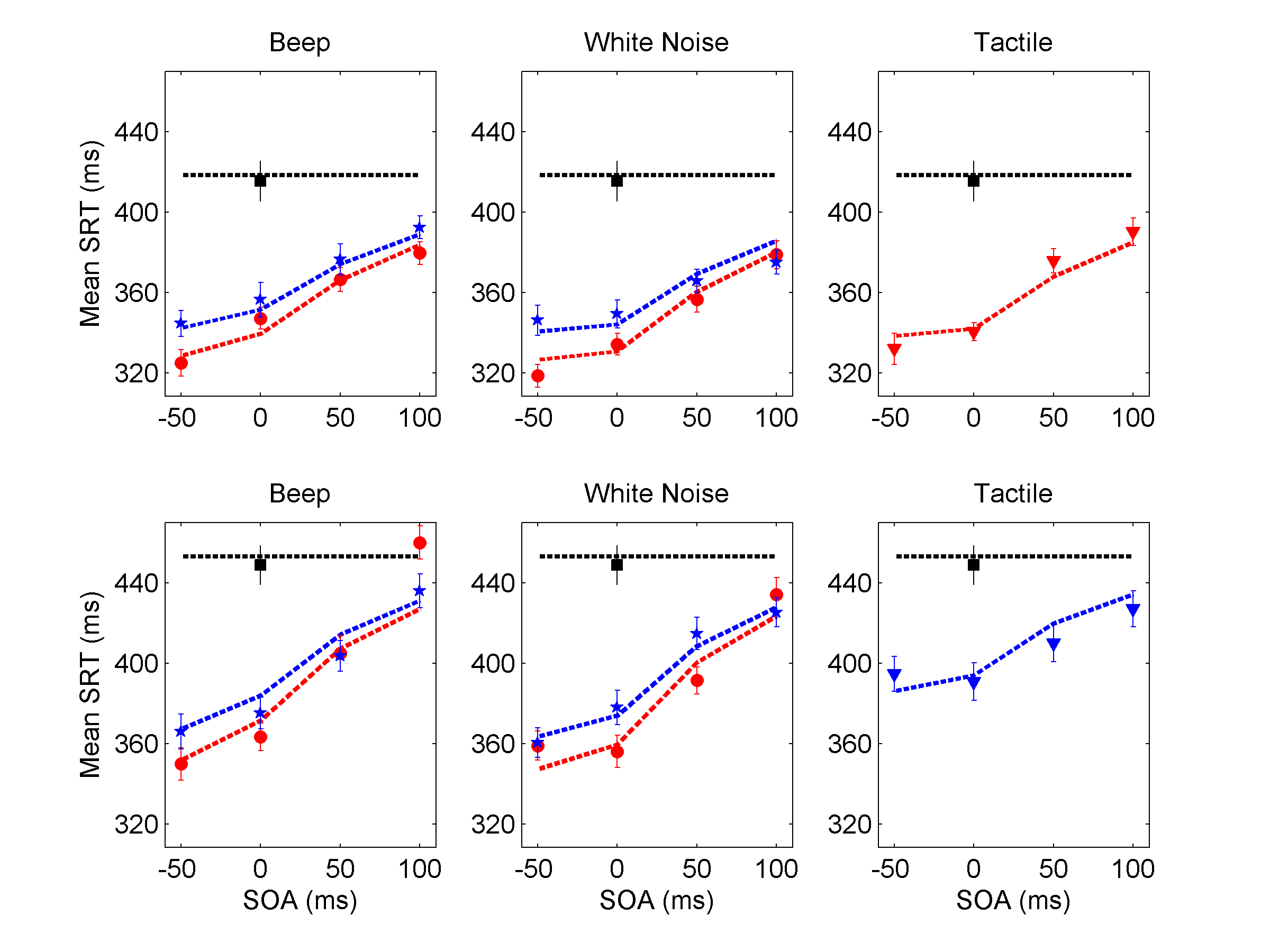

Supplement: Figure S1 — Observed and predicted data for VP1. (TIF) [file pone.0092666.s001.tif]

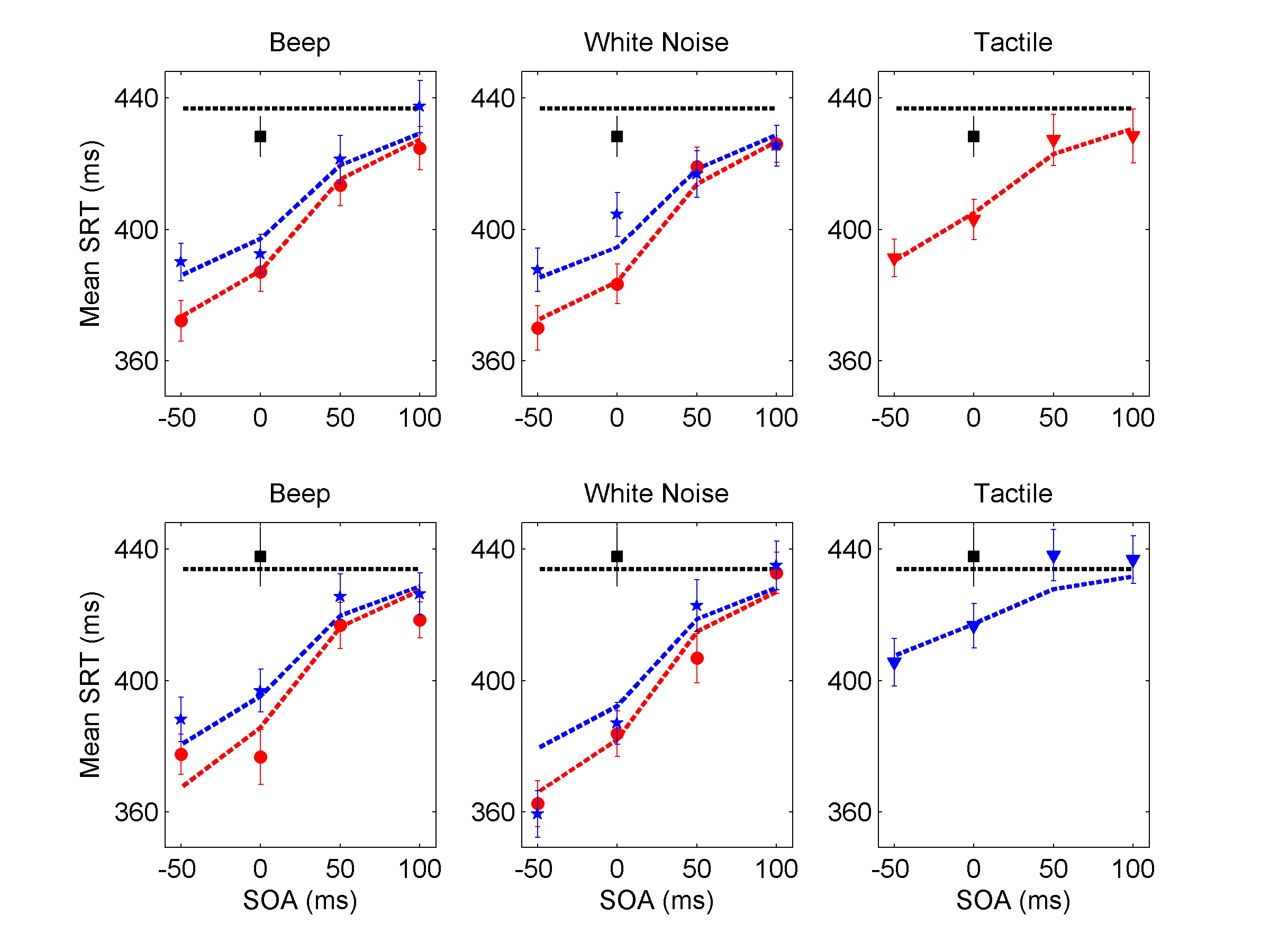

Supplement: Figure S2 — Observed and predicted data for VP2. (TIF) [file pone.0092666.s002.tif]

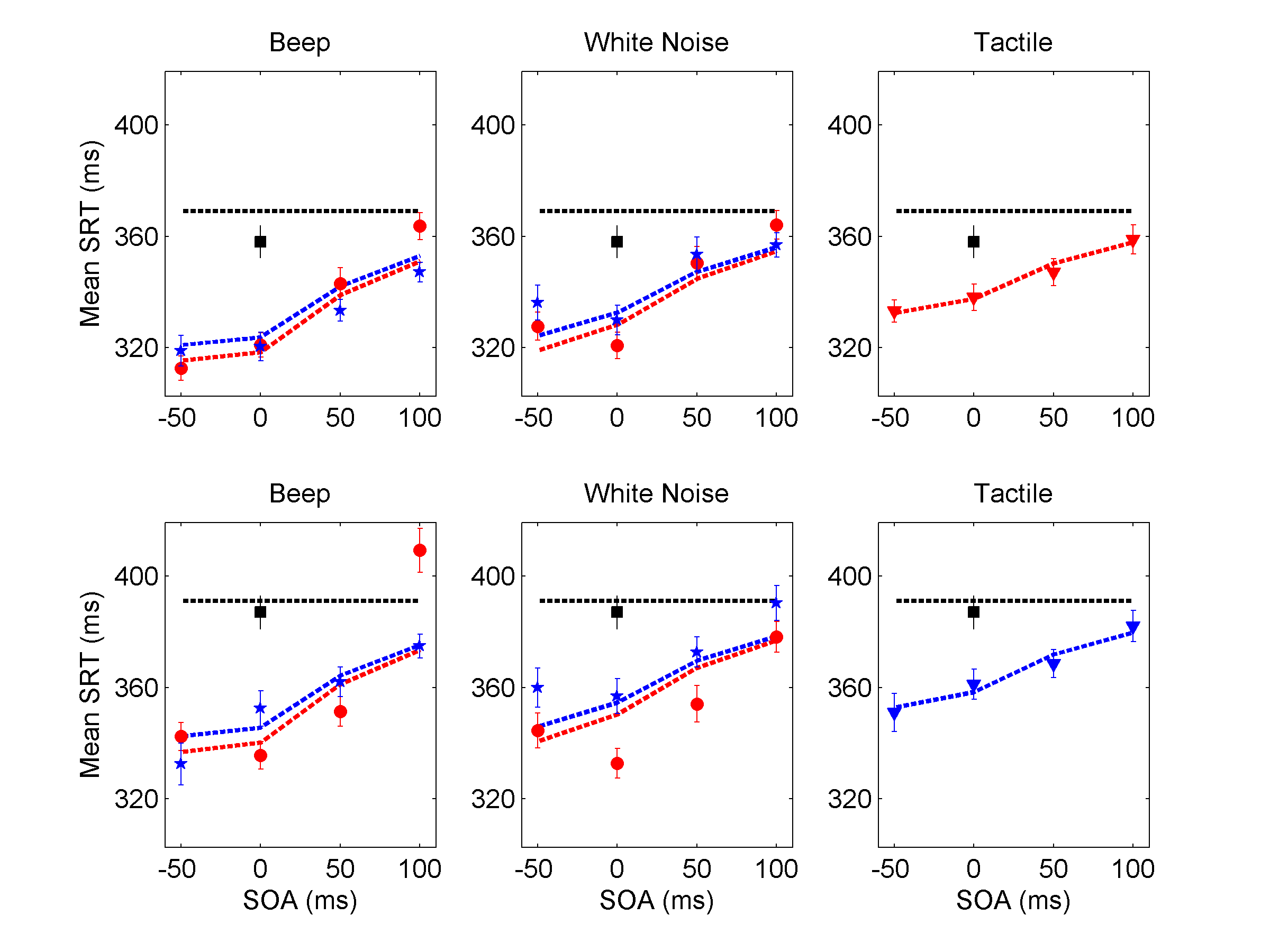

Supplement: Figure S3 — Observed and predicted data for VP3. (TIF) [file pone.0092666.s003.tif]

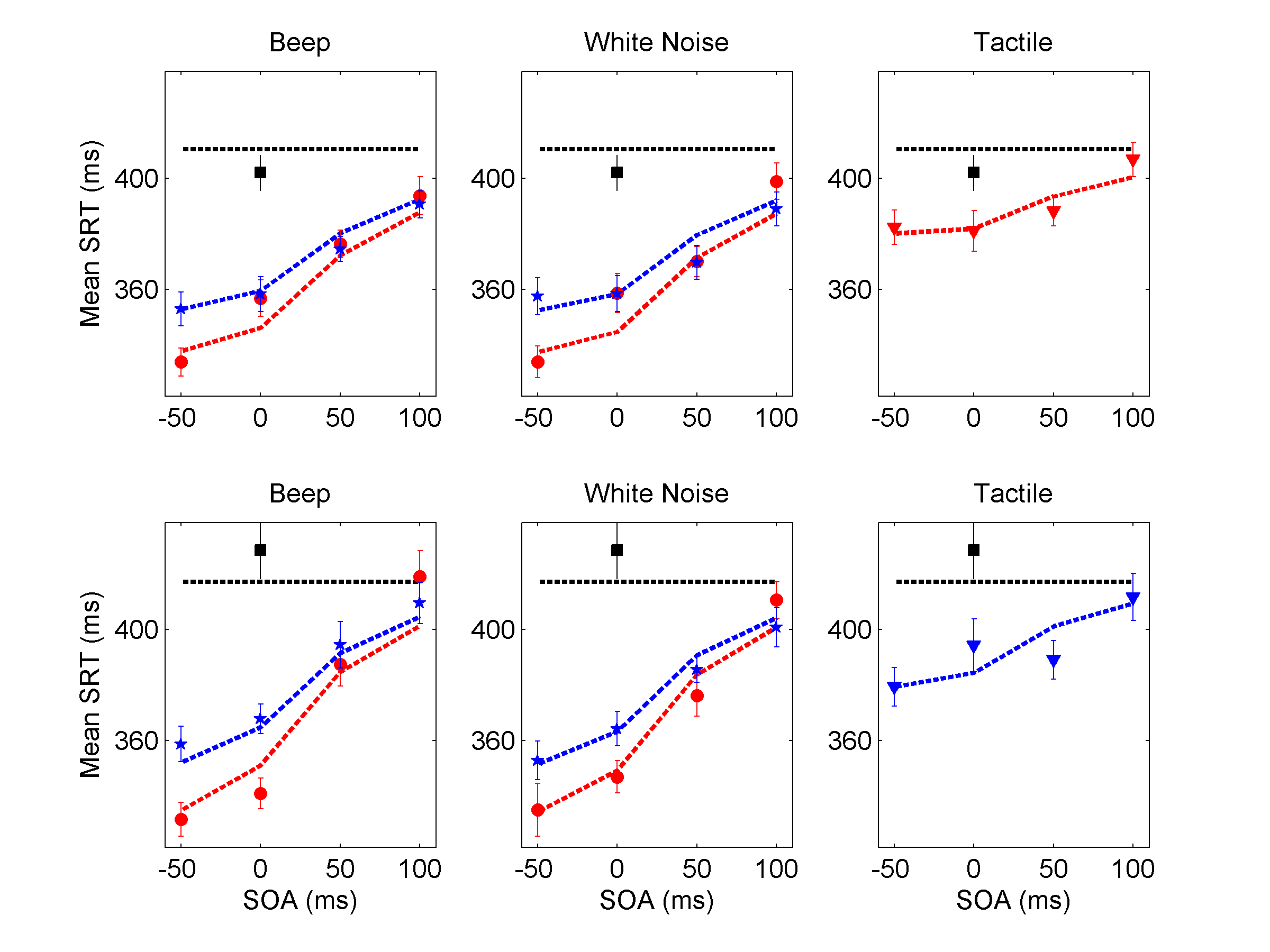

Supplement: Figure S4 — Observed and predicted data for VP4. (TIF) [file pone.0092666.s004.tif]

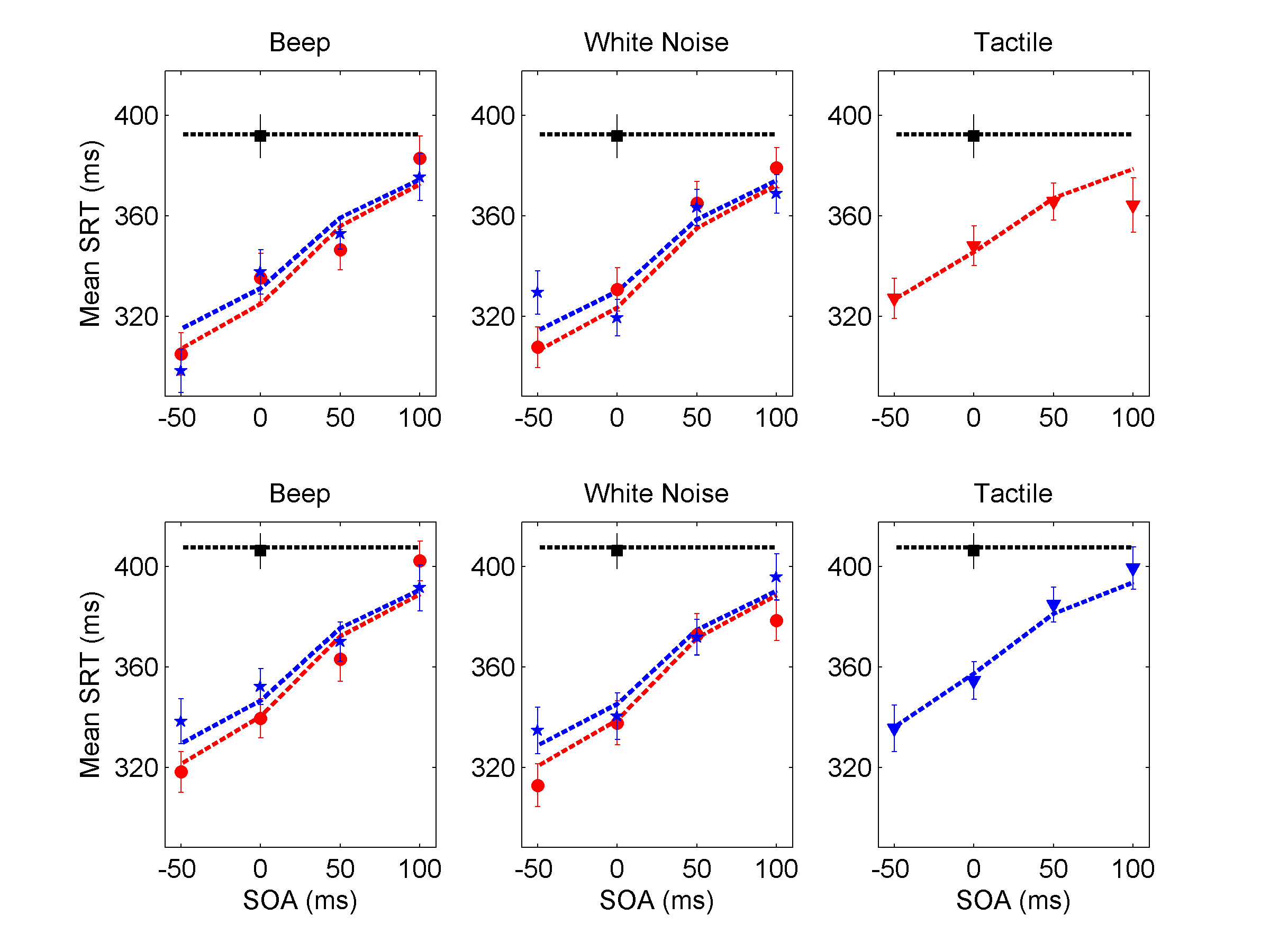

Supplement: Figure S5 — Observed and predicted data for VP5. (TIF) [file pone.0092666.s005.tif]

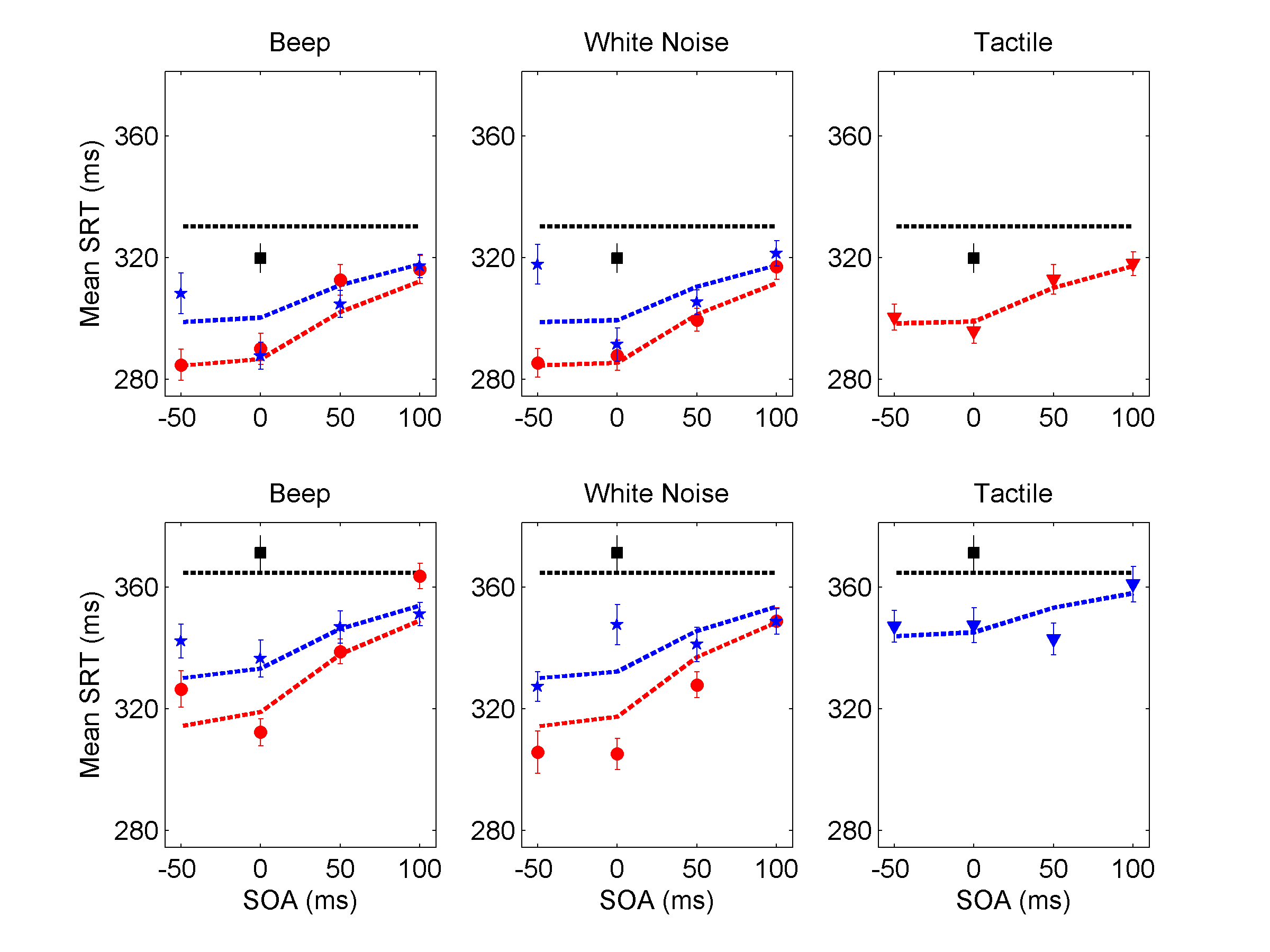

Supplement: Figure S6 — Observed and predicted data for VP6. (TIF) [file pone.0092666.s006.tif]

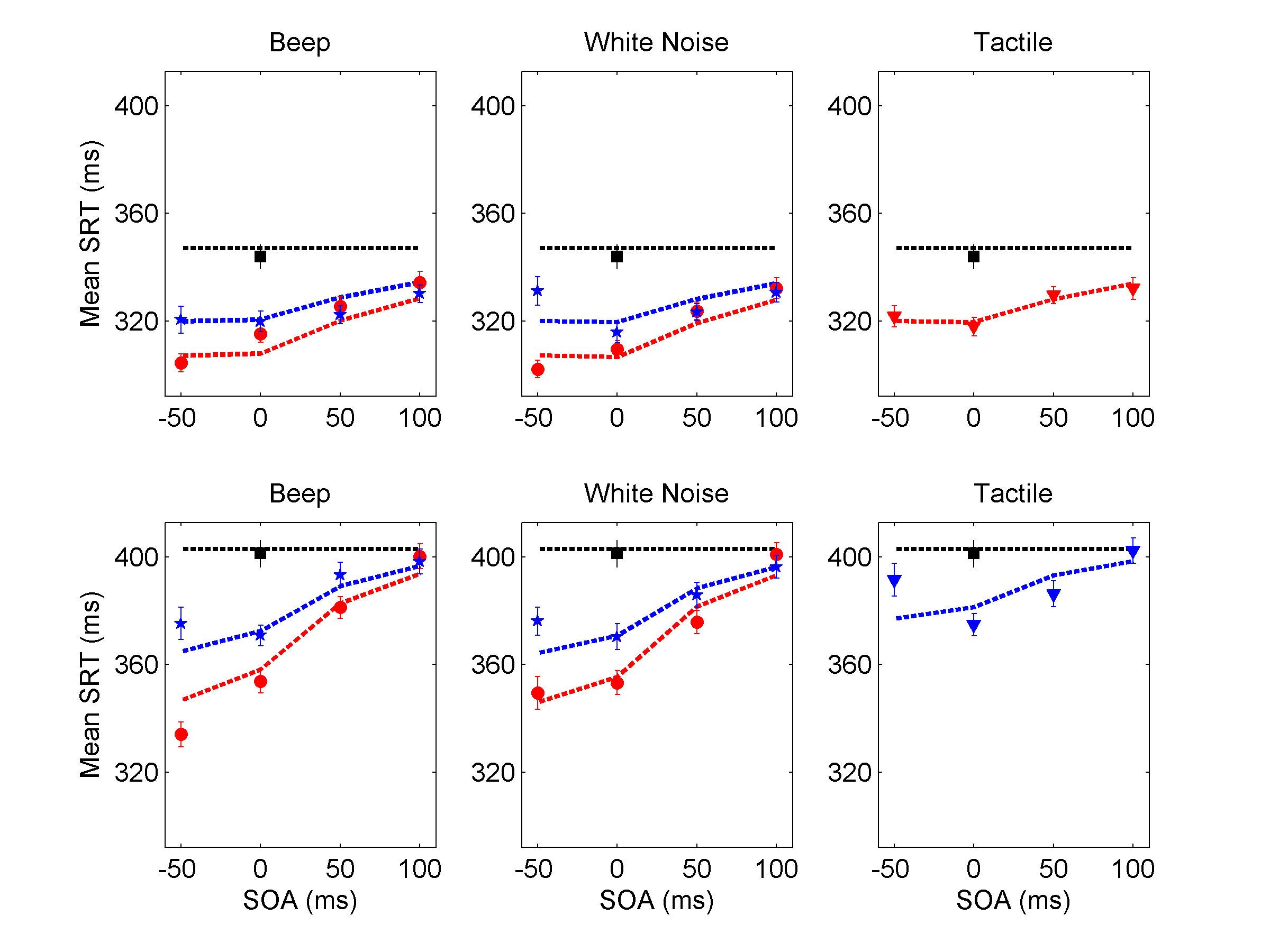

Supplement: Figure S7 — Observed and predicted data for VP7. (TIF) [file pone.0092666.s007.tif]
